# Molecular Verification of New World *Mansonella perstans* Parasitemias

**DOI:** 10.3201/eid2303.161159

**Published:** 2017-03

**Authors:** Lucyane Bastos Tavares da Silva, James Lee Crainey, Túllio Romão Ribeiro da Silva, Uziel Ferreira Suwa, Ana Carolina Paulo Vicente, Jansen Fernandes de Medeiros, Felipe Arley Costa Pessoa, Sérgio Luiz Bessa Luz

**Affiliations:** Fundação Oswaldo Cruz Amazônia Instituto Leônidas e Maria Deane, Amazonas, Brazil (L.B. Tavares da Silva, J.L. Crainey, T.R. Ribeiro da Silva, U.F. Suwa, F.A.C. Pessoa, S.L. Bessa Luz);; Fundação Oswaldo Cruz Instituto Oswaldo Cruz, Rio de Janeiro, Brazil (A.C. Paulo Vicente);; Fundação Oswaldo Cruz Rondônia Laboratório de Entomologia, Rondônia, Brazil (J. Fernandes de Medeiros)

**Keywords:** *Mansonella* parasitemia, molecular verification, New World, Brazil, Amazonas, São Gabriel da Cachoeira, Africa, *Mansonella perstans*, *Mansonella ozzardi*, parasites, parasitic infection, *Culicoides* midges, vector-borne diseases

## Abstract

We obtained ribosomal and mitochondrial DNA sequences from residents of Amazonas state, Brazil, with *Mansonella* parasitemias. Phylogenetic analysis of these sequences confirm that *M. ozzardi* and *M. perstans* parasites occur in sympatry and reveal the close relationship between *M. perstans* in Africa and Brazil, providing insights into the parasite’s New World origins.

*Mansonella perstans* is one of the most prevalent and poorly understood parasites known to cause parasitemias in humans (*1**–**3*). An estimated 114 million persons are infected with *M. perstans* parasites in Africa alone, and *M. perstans* parasitemias have also been repeatedly reported to occur in continental South America (*1**,**2*). In Uganda, *M. perstans* infections and parasitic loads have been shown to map closely with the larval breeding sites of its known vector, the *Culicoides* midge (*1*). Almost nothing is known about the parasites’ epidemiology in continental South America; however, it has been established that simuliids and a diverse range of Ceratopogonid vector species transmit *M. ozzardi* parasites in Latin America (*1*). Thus, it cannot safely assumed that the epidemiology of *M. perstans* in Latin America is particularly similar to its epidemiology in Africa (*1**,**2*).

Like most reports of *M. perstans* in Africa, reports of the occurrence of *M. perstans* in South America have almost always been based on morphologically identified microfilariae observed in blood smears (*1**,**2*). However, in contrast to the situation in Africa, where only 1 parasitemia-causing *Mansonella* parasite occurs, reports of *M. perstans* in South America have been limited to equatorial rainforest regions, where other *Mansonella* parasitemia-causing parasites also commonly occur (*1**–**4*). Therefore, microscopy-based *Mansonella* parasitemia diagnoses in Latin America can be regarded as more prone to error than those made in Africa (*1**–**6*). Conspicuously, *M. perstans* DNA sequences originating outside of Africa have until now been missing, and the relationship between *M. perstans* in Africa and *M. perstans* in the New World has been a mystery (*1*).

By using 3 DNA sequences commonly used in the molecular systematics of filarial parasites (the nuclear internal transcribed spacer 1 [ITS1]–based ribosomal DNA sequence [*7*] and the mitochondrial 12S and cytochrome c oxidase subunit 1 genes [*6*]), we confirmed *M. perstans* microfilariae morphologic identifications made using thick blood smears prepared from persons residing in the village of São Gabriel da Cachoeira, Amazonas state, Brazil. Besides providing verification of *M. perstans* morphologic identifications, the ITS1 sequences generated for this study allowed a phylogenetic analysis with *M. perstans* from Africa. The ribosomal ITS1 *M. perstans* from Brazil clustered with other *M. perstans* ITS1 sequences originating from Africa in a strongly (>94%) bootstrap-supported *M. perstans*–exclusive monophyletic group (Figure). Similarly, *M. ozzardi* ITS1 sequences obtained from parasites from Brazil clustered in another strongly (>97%) bootstrap-supported monophyletic group containing only *M. ozzardi* origin sequences.

**Figure:**
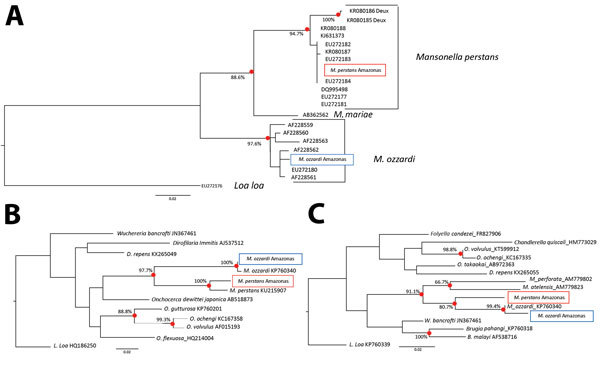
Maximum-likelihood phylogenetic trees showing the relationship between *Mansonella* parasites from Amazon region of Brazil (Amazonas state) and some of their closest relatives. A) Ribosomal internal transcribed spacer 1–based phylogeny. B) Mitochondrial cytochrome c oxidase subunit 1–based phylogeny. C) Mitochondrial 12S-based phylogeny. All 3 trees were prepared by using DNA sequence alignments and PHYLIP version 3.67 (http://evolution.genetics.washington.edu/phylip.html). Black circles indicate significant bootstrap-supported nodes as a percentage of 1,000 pseudoreplicates. Solid boxes indicate *M. perstans* and dashed boxes *M. ozzardi* sequences generated for this study and used in the construction of the displayed trees. Scale bars indicate nucleotide substitutions per site. These sequence have been submitted to GenBank and EMBL (accession nos.: *M. perstans* cytochrome c oxidase subunit 1, LT623909; *M. ozzardi* cytochrome c oxidase subunit 1, LT623910; *M. perstans* 12S, LT623913; *M. ozzardi* 12S, LT623914; *M. perstans* internal transcribed spacer 1, LT623911; and *M. ozzardi* internal transcribed spacer 1, LT623912).

The genetic distance between the ITS1 sequences of *M. perstan*s from Brazil and their closest relatives from Africa is very small (corresponding to <1% divergence across 396 nucleotide positions). From the ITS1-based phylogenetic analysis, the *M. perstans* from Brazil appear to be more closely related to some *M. perstans* in Africa than they are to others. The ITS1 sequences from *M. perstans* previously described as *M. perstans* “deux” (*8*) and originating from Gabon can be observed in a bootstrap-supported cluster forming a sister clade to the bootstrap-supported monophyletic cluster containing the *M. perstans* from Brazil, which also contains sequences originating from Cameroon, Côte d’Ivoire, Equatorial Guinea, Gabon, Mali, and Sierra Leone. Thus, our results suggest that *M. perstans* arrived in Latin America after the standard form of *M. perstans* diverged from the *M. perstans* “deux” form.

Sequences from mitochondrial genes 12S rDNA and cytochrome c oxidase subunit 1 have also been recovered from blood samples in Brazil and used to confirm morphologic and ITS1-based *Mansonella* parasite identifications (*6*). Phylogenetic analysis performed with these mitochondrial gene segments was consistent with our ITS1 analysis and also suggest that *M. perstans* arrived in Latin America very recently (Figure). In addition to verifying that South America does indeed have the conditions to support *M. perstans* and providing a useful reference for vector incrimination and other epidemiologic studies, our findings have also provided insights into the origin of the *M. perstans* parasite in South America. Given how similar our findings are to those obtained when *Onchocerca volvulus* parasite mitogenomes from Latin America and Africa have been compared, they suggest that *M. perstans*, like *O. volvulus*, probably arrived in Latin America as a consequence of the slave trade (*9**–**10*).
